# Promiscuity and preferences of metallothioneins: the cell rules

**DOI:** 10.1186/1741-7007-9-25

**Published:** 2011-04-28

**Authors:** Andrew W Foster, Nigel J Robinson

**Affiliations:** 1 Biophysical Sciences Institute, Department of Chemistry, School of Biological and Biomedical Sciences, Durham University, DH1 3LE, UK

## Abstract

Metalloproteins are essential for many cellular functions, but it has not been clear how they distinguish between the different metals to bind the correct ones. A report in *BMC Biology *finds that preferences of two metallothionein isoforms for two different cations are due to inherent properties of these usually less discriminating proteins. Here these observations are discussed in the context of the cellular mechanisms that regulate metal binding to proteins.

See research article: http://www.biomedcentral.com/1741-7007/9/4

## 

Metals are essential to the structure and function of many proteins, from DNA-binding zinc fingers to respiratory proteins that require iron or copper. It has been estimated that nearly half of all enzymes are metalloproteins [1], although vast numbers of metalloproteins may remain uncharacterized [2]. A fundamental question about all such proteins is what determines which metals they bind. In some cases metals are delivered to the metalloproteins by specialized metallochaperones. But for most metalloproteins, a critical factor is thought to be the availability of the appropriate metal species in the buffered pools in the cell. These vital buffered metal pools need to be somehow measured.

Metallothionein proteins provide cysteine thiolate ligands for metals and constitute a part of the metal-buffer in cells, both for storing biologically important metals and for sequestering toxic ones. These proteins usually show similar preferences to each other in the metals that they bind. In a recent paper in *BMC Biology*, Dallinger and colleagues (Palacios *et al. *[3]) report investigations on two metallothionein isoforms of snails that, despite having an identical number and arrangement of cysteine residues, seem to differ in their choice of copper or cadmium. The authors conclude that a high degree of metal selectivity is conferred by the inherent properties of the proteins.

## Copper, cadmium and the biology of snail metallothioneins

The two metallothionein isoforms studied by Palacios *et al. *[3] are HpCuMT and HpCdMT from the Roman snail *Helix pomatia*. HpCuMT is constitutively expressed in snails in a specialized molluscan cell type, the rhogocyte, which is the site of synthesis of the copper protein hemocyanin [3]. As its name suggests, HpCuMT has always been recovered from the snail tissue as a homometallic copper protein. In contrast, HpCdMT is induced in many cell types in snails exposed to cadmium, and is recovered as a homometallic cadmium protein.

To find out whether the metals acquired by these proteins are due to the differential availability of the two metals at the site of synthesis of the metallothioneins or due to the inherent properties of the proteins, Palacios *et al. *expressed the two metallothioneins in *Escherichia coli *and yeast cells under conditions of varying metal exposure. In the presence of elevated copper and low oxygen, they recovered HpCuMT from *E. coli *as a homometallic copper protein whereas under the same conditions HpCdMT was recovered as a mixed species containing zinc (this protein is thought normally to buffer zinc but to bind cadmium after cadmium intoxication) as well as copper [3]. Conversely, when HpCdMT was expressed in *E. coli *enriched with either cadmium or zinc, homometallic, fully populated cadmium or zinc forms were recovered, although analogous expression of HpCuMT gave variable occupancy with cadmium or zinc [3]. The *H. pomatia *proteins also rescued sensitivity to cadmium or to copper in yeast mutants with metal sensitivities that matched the metals selected by the respective metallothioneins. Retention of metal preferences in heterologous hosts argues that selectivity resides in the proteins. However, a heterologous environment still contains other proteins contributing to the buffering of metals, and these data do not necessarily mean, for example, that HpCdMT binds cadmium and/or zinc more tightly than copper. Rather, the data reflect the metal preferences of HpCdMT relative to other components of the mixed metal buffers of the organisms and cell types.

## What determines the metal preferences of proteins?

**Figure 1:**
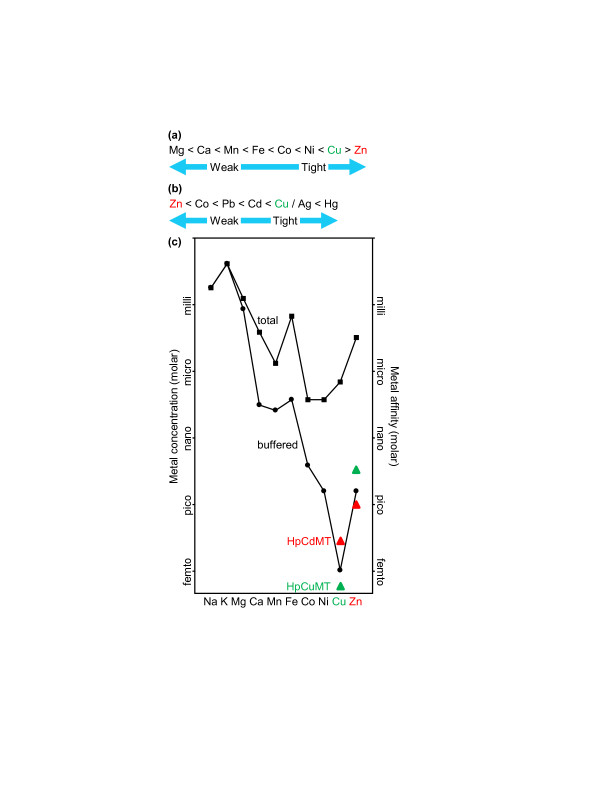
**Universal orders of metal preference and proposed buffered metal concentrations in cells**. **(a) **The Irving-Williams series provides a preferred binding order for divalent metals. **(b) **An order of preference for thiolate-containing ligands of some nonessential, and some essential, metals. **(c) **How the occupancy of HpCdMT and HpCuMT might relate to the buffered concentrations of metals in a hypothetical cell. The total concentration of each metal in a cell (squares) is many orders of magnitude greater than the buffered concentration (circles), with the exception of bulk solutes such as sodium and potassium. The values have been adapted from a proposal of da Silva and Williams [4]. Proteins with affinities tighter than the buffered concentrations can acquire metals. HpCuMT (green triangles) is suggested to have an affinity for copper slightly tighter than the buffered concentration of copper found in many cell types, whereas HpCdMT (red triangles) is suggested to have a copper affinity too weak to compete effectively with the copper buffers of at least some cell types. In this scheme the situation is reversed for zinc. There is evidence that the relationship between the *H. pomatia *metallothioneins and buffers for non-cognate metals depart from this scheme in some cell types.

Because proteins are flexible, they offer imperfect steric selection between metals. This is especially true of nascent proteins before folding. The affinities of proteins for metals are influenced by universal orders of preference, which for biologically essential divalent metals includes the Irving-Williams series (Figure 1a), which ranks the relative stability of complexes formed with each metal ion [4]. (Monovalent copper also forms tight associations with proteins, particularly when the ligands are cysteine thiolates, as in metallothioneins.) Several nonessential toxic metals, including mercury, cadmium and silver, also form tight complexes with thiolates, obeying an order of preference listed in Figure 1b[3]. Under the strictures of such affinity series how do large numbers of proteins become populated with less competitive metals such as magnesium and manganese, avoiding displacement by more tightly binding metals such as copper? Part of the answer is that the buffered concentrations of metals are controlled in cells in such a way that the most competitive metals are bound and buffered to the lowest available concentrations. This is illustrated by the predominant manganese protein and the predominant copper protein in the periplasm of a cyanobacterium [5]. These proteins, MncA and CucA, respectively, have identical sets of metal ligands and similar cupin folds. Moreover, the manganese protein MncA has a 10,000 times greater preference for the wrong metal, copper, than for manganese. However, whereas the copper protein folds after membrane translocation, MncA folds in the cytosol before translocation. Therefore, the cytosol must be a protected environment where the ratio of buffered copper to buffered manganese is less than 1:10,000, at least at the site where MncA folds. Once the protein has correctly enfolded manganese, the metal becomes kinetically entrapped and safe from replacement by more competitive metals such as copper [5]. The folding location thus overrides the metal-binding preference of MncA to dictate metal occupancy. Precise control of the ratios of buffered metals available to proteins at folding is thus crucial to ensure binding of the correct inorganic elements. Computational studies similarly conclude that in biological systems, in the absence of metallochaperones, the specificity of protein ligands for metals depends mainly on the local abundances of metals [6].

## Binding affinity, competition and control of metal availability in cells

The availability of metals in cells is thought to be regulated by the actions of DNA-binding metal sensors that control the expression of genes encoding proteins of metal homeostasis, including metal-buffering proteins such as metallothioneins [1]. These sensors act to maintain the buffered concentrations of metals within limits determined, at least in part, by their own metal affinities [7]. Under such a regime the metal affinities of *E. coli *metal sensors will influence metal occupancy of snail metallothioneins when expressed in *E. coli*. The copper affinity of the *E. coli *copper sensor CueR, relative to the zinc affinities of the zinc sensors ZntR and Zur, suggests that copper is buffered to an even lower concentration than zinc [8,9] in *E. coli*. A protein is expected to gain access to a given metal only if the affinity of the protein for that metal is tighter than the buffered concentration. Thus, HpCuMT is predicted to have an affinity for copper tight enough to compete with other molecules that buffer copper in rhogocytes, and also in *E. coli *and yeast. In contrast, HpCdMT is predicted to have an affinity for copper that is less able to compete with these other buffers (Figure 1c).

Measurement of the absolute metal affinities of proteins has been surprisingly challenging, with many erroneous values in the literature [10]. The copper, cadmium and zinc affinities of *H. pomatia *metallothioneins remain to be measured, but the scheme in Figure 1c suggests that HpCdMT and HpCuMT both have tighter affinities for copper than for cadmium or zinc, in accordance with the series in Figure 1a,b. Subtle differences between the two sequences must nonetheless give HpCuMT the tighter copper affinity of the two, as when both are expressed in the presence of excess copper in *E. coli*, only HpCuMT becomes fully populated with copper [3]. Notably, even after growth of *E. coli *in excess copper, recombinant HpCuMT was partly occupied with zinc unless the cells were also depleted of oxygen. Perhaps copper is buffered to a slightly lower concentration in aerobic *E. coli *than in rhogocytes, or perhaps the *E. coli *copper pool in aerobic conditions is swiftly depleted by overexpression of HpCuMT. There is no known demand for copper in the *E. coli *cytosol, although there is emerging evidence that periplasmic copper proteins are supplied with copper through export from the cytosol.

It is hypothesized that the metals that occupy proteins and are critical to their function could be regulated according to cell type by adjusting the buffered metal concentrations to different settings in different cell types. HpCuMT itself contributes to the copper buffer in rhogocytes, implying that copper is buffered more tightly in these cells than in other cells, perhaps to withhold copper more effectively from metalloproteins other than hemocyanin. Technologies are being developed to measure the elusive availabilities of metals to nascent proteins. The mechanisms that maintain these buffered concentrations underlie much of biological catalysis.
